# Neural activity tied to reading predicts individual differences in extended-text comprehension

**DOI:** 10.3389/fnhum.2013.00655

**Published:** 2013-11-06

**Authors:** Julia A. Mossbridge, Marcia Grabowecky, Ken A. Paller, Satoru Suzuki

**Affiliations:** ^1^Department of Psychology, Northwestern UniversityEvanston, IL, USA; ^2^Interdepartmental Neuroscience Program, Northwestern UniversityEvanston, IL, USA

**Keywords:** reading comprehension, EEG/ERP, machine learning applied to neuroscience, current source density, working memory

## Abstract

Reading comprehension depends on neural processes supporting the access, understanding, and storage of words over time. Examinations of the neural activity correlated with reading have contributed to our understanding of reading comprehension, especially for the comprehension of sentences and short passages. However, the neural activity associated with comprehending an extended text is not well-understood. Here we describe a current-source-density (CSD) index that predicts individual differences in the comprehension of an extended text. The index is the difference in CSD-transformed event-related potentials (ERPs) to a target word between two conditions: a comprehension condition with words from a story presented in their original order, and a scrambled condition with the same words presented in a randomized order. In both conditions participants responded to the target word, and in the comprehension condition they also tried to follow the story in preparation for a comprehension test. We reasoned that the spatiotemporal pattern of difference-CSDs would reflect comprehension-related processes beyond word-level processing. We used a pattern-classification method to identify the component of the difference-CSDs that accurately (88%) discriminated good from poor comprehenders. The critical CSD index was focused at a frontal-midline scalp site, occurred 400–500 ms after target-word onset, and was strongly correlated with comprehension performance. Behavioral data indicated that group differences in effort or motor preparation could not explain these results. Further, our CSD index appears to be distinct from the well-known P300 and N400 components, and CSD transformation seems to be crucial for distinguishing good from poor comprehenders using our experimental paradigm. Once our CSD index is fully characterized, this neural signature of individual differences in extended-text comprehension may aid the diagnosis and remediation of reading comprehension deficits.

## Introduction

The most enduring model of reading is the “simple model” first proposed by Gough and Tunmer (1986). The simple model holds that effective reading requires two fundamental steps: word decoding and language comprehension (Hoover and Gough, 1990; King and Kutas, 1995; Rapp et al., 2007; Ferstl et al., 2008). Word decoding describes the process of making correct pairings between visual word forms and their associated sounds. Language comprehension describes the process of accessing the meanings of spoken or written words, integrating these words into meaningful discourses, and maintaining the apprehended meaning over time.

Much effort has been made to understand the neural mechanisms of language comprehension. Language networks in the human brain have been identified by monitoring time-averaged metabolic brain activity using neuroimaging methods such as functional magnetic resonance imaging (fMRI) and positron emission tomography (PET) (see Ferstl et al., 2008 for a meta-analysis). Investigations of the neural underpinnings of reading have also relied on the excellent temporal resolution of methods such as electroencephalography (EEG) because word decoding and language comprehension occur in temporal stages that overlap one another during reading. In particular, experiments that use a stimulus-locked averaging approach to produce event-related potentials (ERPs) have revealed separable components related to reading, some of which are more closely related to decoding, others to comprehension. One of the most well-established components related to decoding is the N170, a component that is usually negative-going (depending on the reference electrode), peaking at about 170 ms after word onset (for review, see Maurer and McCandliss, 2008). Word decoding efficiency is correlated with the difference between left-lateralized N170 responses to words vs. non-words (Maurer et al., 2007; Coch and Mitra, 2010). Perhaps the best-established ERP component related to language comprehension is the N400, which is negative-going and peaks at about 400 ms after word onset over the central-parietal scalp region (Lau et al., 2008). The amplitude of the N400 is less negative when a word is predictable from its global or local context (as compared to the more negative response for unpredictable words), suggesting that the N400 reflects the access and/or integration of word meaning (Kutas and Hillyard, 1980, 1984; Kutas and Federmeier, 2000).

This progress in using ERPs to characterize the neural activity related to reading comprehension has been made despite a notable limitation. Most ERP (also fMRI and PET) studies of reading comprehension have used a few (1–5) sentences or single paragraphs as reading materials rather than extended texts (Kutas and Hillyard, 1980; St. George et al., 1994; King and Kutas, 1995; VanPetten et al., 1997; Kutas and Federmeier, 2000; Ferstl et al., 2008; Lau et al., 2008). Meanwhile, the comprehension of an extended text is likely to engage processes that are not substantially engaged while reading a few sentences or a short paragraph. For example, the comprehension of an extended text is likely to place heavy demands on verbal working memory (Daneman and Carpenter, 1980; Daneman and Merikle, 1996; Swanson and Alexander, 1997; Cain et al., 2004; Swanson et al., 2006; Cutting et al., 2009; Chein and Morrison, 2010; Dahlin, 2011), to require processes that resolve the build-up of proactive interference (Lustig et al., 2001; Bunting, 2006), and to include higher-order processes for grasping ideas that span many paragraphs. Although syntactic and semantic manipulations within a sentence have been used to understand the neural processes involved in reading at the level of sentences (e.g., King and Kutas, 1995), it has not been clear how to examine neural activity associated with the comprehension of extended texts. For example, it is unclear how the content of an extended text should be manipulated so that this manipulation would distinguish the neural activity specific to comprehension from the neural activity related to word-level processing.

**Figure 1:**
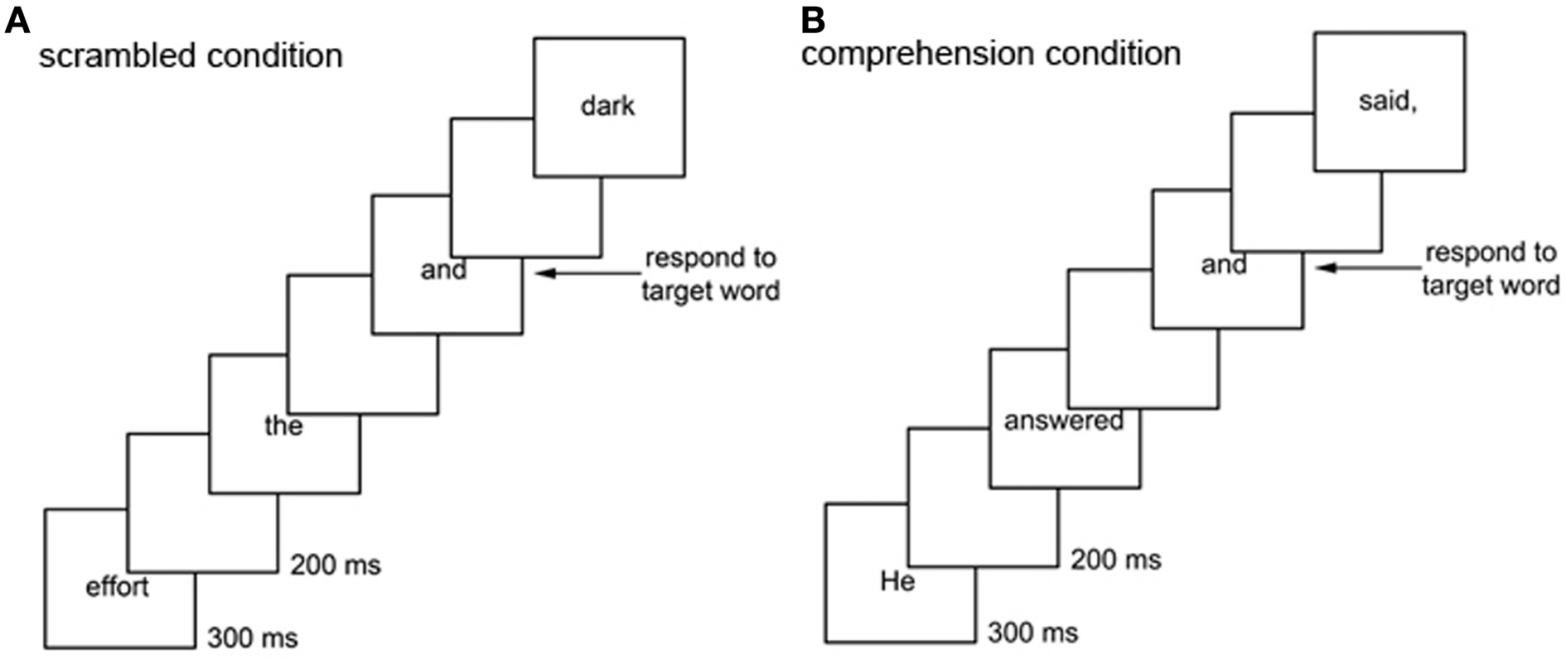
**Scrambled and comprehension conditions.** In the scrambled condition **(A)**, a story was presented in a scrambled order one word every 500 ms, and participants were asked to perform the target-word detection task: pressing a mouse button every time they saw the word “and.” In the comprehension condition **(B)**, the same story was presented in its original order one word every 500 ms, and participants were asked to perform the target-word detection task and also to follow the story in preparation for a comprehension test. Condition order was counterbalanced across participants.

In the current study, we attempted to overcome this limitation by using an extended text (over 1100 words), a word-detection task, a comprehension test given at the end of reading the entire text, an individual-differences approach, and a pattern-classification algorithm, all combined to allow us to identify the spatiotemporal pattern of ERPs specific to extended-text comprehension. Specifically, to isolate the neural activity tied to language comprehension (rather than word decoding and access), we compared electrophysiological responses to the same target word (“and”) between two conditions (Figure 1). In one condition, which we call the *scrambled condition*, we presented the words of a story visually, one word at a time in a scrambled order, and we asked participants to press a button whenever the target word appeared (Figure 1A). In the other condition, which we call the *comprehension condition*, we presented the same words but in the original order in which they appear in the story, and we asked participants to perform the same target-word detection task (Figure 1B). In the comprehension condition we also asked participants to simultaneously try to follow the story in preparation for a comprehension test. We selected “and” as the target word because it is a function word joining two words or phrases, so that ERPs in response to it are likely to reflect the integration and storage processes required for comprehension in the context of a story, but not in the context of a random sequence of words. Also, the word “and” naturally appears relatively frequently in almost any extended English text to provide an appropriate signal-to-noise ratio for computing reliable ERPs; at the same time, because “and” is a highly familiar word and its occurrence is rare (2.4% in our extended text) the target-detection task caused minimal interference with reading. Thus, to extract comprehension-related processes beyond word-level processing, we used the difference waves computed as the difference in ERPs to the target word between the comprehension and scrambled conditions.

To further distinguish the processes specifically tied to the comprehension of extended text beyond the comprehension of individual sentences and paragraphs, we determined the spatiotemporal pattern of the ERP difference waves (between the comprehension and scrambled conditions) that predicted individual differences in comprehension based on an evaluation of sustained understanding throughout the entire text. We hypothesized that readers with good reading comprehension should engage comprehension-specific neural processes more effectively than readers with poor reading comprehension, so that a specific spatiotemporal pattern of the difference waves would distinguish good vs. poor extended-text comprehenders. We attempted to identify this comprehension-specific pattern of electrophysiological activity in a data-driven manner by letting an automated non-linear pattern-classification algorithm determine the critical scalp sites and time points that most accurately predicted individual differences in comprehension.

In order to characterize the critical electrophysiological activity with high spatial resolution, instead of examining raw ERPs (as electrical potentials) we examined their second spatial derivative [known as the current-source-density (CSD) transform, or CSD; e.g., Hjorth, 1980; Kayser and Tenke, 2006; Tenke and Kayser, 2012]. CSD-transformed ERPs indicate the spatial distribution of charge density (implying current sources and sinks), and the transformation increases spatial resolution by reducing the influences from volume conduction (see Methods). Our results suggest that CSD transformation (and potentially other spatial-resolution enhancing transformations) may be crucial for characterizing electrophysiological activity that distinguishes good from poor comprehenders.

## Materials and methods

### Participants

Twenty-eight right-handed college and graduate students (18–29 years; 17 female) gave their written informed consent to participate in the study following the procedures approved by Northwestern University's Institutional Review Board. All had normal or corrected-to-normal vision and normal hearing with no history of neurological disorders. We required normal hearing because individuals who have had lifelong hearing disorders may process words differently than do typically hearing individuals (e.g., Kutas et al., 1987).

### Scrambled and comprehension conditions

The text consisted of the first 1182 words of the first chapter of *Doctor Pascal* by Emile Zola (Lexile score 1170; see Appendix). No participant had previously read this text. The words were presented one at a time with accompanying punctuation marks on a monitor for 300 ms, each with an inter-word interval of 200 ms. Note that we used a relatively fast presentation of the words to approach the average speed of prose reading for college students (one word per ~200–250 ms; Carver, 1992) while leaving time after word presentation to record EEG responses to each word. Visual angles ranged from 0.49 to 0.73° (vertical; range due to variable letter height) and 0.61–3.64° (horizontal; range due to word length). The words were white (76.8 cd/m^2^) and presented centrally on a black background (4.9 cd/m^2^). Participants performed a scrambled and a comprehension condition, each of which lasted about 10 min. The order of the conditions was counter-balanced across participants. We used Presentation software (version 11.0, Build 04.25.07, www.neurobs.com) running on a Dell Optiplex Gx620 (Intel processor running Windows XP Professional 2002) to present stimuli (on a 21″ color monitor with 1024 by 768 resolution at a refresh rate of 60 Hz) and to record behavioral responses.

In the scrambled condition, participants viewed the words from the story in a randomized order and were asked to press a mouse button with their right hand as soon as they saw the word “and” in the text. This target word appeared 28 times. Each participant received a different randomized order, making it unlikely that any specific feature of a randomized order contributed to our results. Nonetheless, we took measures to ensure that rare accidental repetitions of the target word in the scrambled condition did not contribute to our results (see Electrophysiological Recordings).

In the comprehension condition, the words were presented in the order of the original text and participants were asked to perform the target-word detection task while also comprehending the story. Immediately after the comprehension condition, participants took a multiple-choice comprehension test consisting of four questions, each with four possible answers (See Appendix). Participants were told that any number of the four answers could be correct for each question, that they should circle all correct answers, and that for each question at least one answer was correct. Questions were answerable by reading and comprehending the text; no general knowledge questions were included (Keenan and Betjemann, 2006; Keenan et al., 2008). Each question was scored as correct if and only if all of the correct answers and none of the incorrect answers were selected out of the four choices. Thus, the maximum possible comprehension score was 4, the minimum possible comprehension score was 0, and the score expected by chance was less than 1 (expectation value = 0.27 questions correct by chance). Across our participants, the mean number of correctly answered questions was 2.6 and the median was 3. Poor comprehenders were defined as individuals who received scores of 0–2 on the test (*N* = 13), and good comprehenders were defined as individuals who received scores of 3–4 (*N* = 15). It is important to note that based on our scoring method, the probability of getting two or more questions correct by chance was 0.024 (1–_4_C_0_[1–1/15]^4^–_4_C_1_[1/15][1–1/15]^3^; each denominator is 15 rather than 16 because participants were told that at least one of the four choices was correct for each question; i.e., not selecting any choice was not an option) and the probability of getting three or more questions correct by chance was 0.0011 (_4_C_3_[1/15]^3^[1–1/15]+_4_C_4_[1/15]^4^). In other words, it was more than 20 times as difficult (probabilistically) to score 3 or higher as to score 2 or higher. Thus, our division of good and poor comprehenders reflected a large difference in comprehension performance.

Classical music (Beethoven's *Moonlight Sonata*) was played quietly in the background in both conditions, a practice that is often used during EEG recording to help participants relax and to reduce muscle activity artifacts (Luck, 2005). Because the same background music was played in both conditions, it is unlikely that the music would have confounded comparisons between conditions.

### Electrophysiological recordings

We recorded EEG with a 64 + 8 active-electrode Biosemi system at a 1024-Hz sampling rate, and data were re-referenced offline to an external electrode at the nose using standard recording procedures, including rejection of blinks. Electrooculographic (EOG) activity was monitored using three facial electrodes, one placed lateral to each eye and one placed beneath the left eye. EEG and EOG data were band-pass filtered from 0.1 to 100 Hz. Each epoch was baselined by subtracting the mean of the first 50 ms of activity after the onset of the target word “and” from each time point in the 500-ms epoch. We used a post- rather than pre-stimulus-onset baseline because the relatively rapid word presentation rate that we used to approximate normal reading speed meant that the response to the previous word was not complete before the presentation of the next word. For similar reasons, post-stimulus-onset baselines have been used in other ERP studies of language processing (e.g., Friederici et al., 1999; Phillips et al., 2005). Note that although post-stimulus-onset baselines could potentially change the amplitudes of the resulting ERP components if the baseline is taken during a positive or negative peak, inspection of raw ERPs (data prior to baseline correction, not shown) suggested that use of the 0–50 ms post-stimulus-onset baseline did not substantially alter any components, as there was minimal activity during this time period.

For each condition (comprehension condition and scrambled condition) we averaged ERPs to the target word “and” for the trials on which it was correctly detected. To reduce potential effects of repetition priming (though such effects should be negligible with very rare targets), we excluded a target trial if any of the four previous trials was also a target trial. Across participants, 3.7% of the target trials in the comprehension condition and 4% of the target trials in the scrambled condition were removed based on these constraints.

### CSD transformation of ERPs

For each participant in each condition, we averaged the remaining artifact-free EEG response waveforms across trials to obtain ERPs, and these ERPs were transformed into CSDs using CSDtoolbox Version 1.1 (Kayser, 2009). We used a CSD transformation for two reasons. First, it provides a reference-independent measure of radial current flow at the scalp (Kayser and Tenke, 2006), making it straightforward to compare our results to future experiments that could potentially use different reference locations. Second, by performing a Laplacian operation (taking the second spatial derivative), CSD transformation minimizes the influence of volume conduction on the recorded signal, thereby sharpening spatial resolution (Hjorth, 1980; Kayser and Tenke, 2006; Tenke and Kayser, 2012). For comparison to traditional ERPs, we also present data from nose-referenced ERPs that are not CSD transformed in the Appendix. Potential relationships between our CSD-based electrophysiological index of extended-text comprehension and two traditional ERP components, the P300 and N400, are also discussed in the Appendix.

### Pattern classification

We used the Matlab 2011b “treebagger” implementation of the non-linear random-forest algorithm to classify good vs. poor reading comprehenders based on their difference-CSDs between the scrambled and comprehension conditions. To obtain the difference-CSDs, we computed the time-point-by-time-point difference between the CSD-transformed ERPs obtained in the scrambled and comprehension conditions (scrambled minus comprehension) for each of the 64 scalp sites and for each participant. We binned these difference-CSDs into eight 100-ms averages overlapping by 50 ms (e.g., 50–150, 100–200, 150–200 ms, etc.). Note that some temporal binning is always necessary; even classification based on “non-binned” time points assumes a bin size equal to the sampling rate. As for any analysis relying on binned data, if the bin is too large, important features may be missed and aliasing can occur. Conversely, if the bin is too small, it is difficult to isolate neural processes that operate at a longer time scale or that are less time-locked to a stimulus. We selected a bin size of 100 ms because we made the assumptions that ERP components attributed to high-level comprehension processes are not as time-locked to stimulus onset as sensory components are, and therefore may be apparent only when using a relatively large bin size. It has also been shown that the sampling rate of visual attention is about 10–15 Hz (e.g., Simpson et al., 2005; VanRullen et al., 2005; Mathewson et al., 2011), commensurate with our 100-ms bin size.

We applied the pattern-classification algorithm (henceforth called the classifier) separately for each time bin to determine how well participants could be classified into good vs. poor reading comprehenders based on the electrophysiological information in each time bin. We ran the classifier in two stages to identify the scalp site(s) most relevant to reading comprehension. In the first stage, we fed difference-CSDs (for the primary analysis, but also fed other features of the ERPs in additional analyses; see below) from all 64 scalp sites to the classifier to allow it to develop an ensemble of 300 decision trees, each of which accurately classified a randomly selected 65% of the participants into good vs. poor reading comprehenders based on different subsets of the 64 scalp sites (for details on the classification algorithm, see for instance (Breiman, 2001; Liaw and Wiener, 2002; Goldstein et al., 2010). This process yielded the “weight” of each of the 64 scalp sites, indicating how substantially each site contributed to classification. In the second stage, we re-ran the classifier, but using data from only the 10 scalp sites with the highest weights. We used this “titration” process to look for the critical electrophysiological information that is localized within a relatively small number of scalp sites. We again allowed the classifier to develop an ensemble of 300 decision trees each of which classified a randomly selected 65% of the participants into good and poor reading comprehenders based on different subsets of the 10 scalp sites. Success of this process indicated that the 10 sites provided sufficient electrophysiological information for classification. However, it is not surprising for the classifier to develop an optimum ensemble of decision trees for a given set of data. If the classifier captures a reliable electrophysiological pattern that generally discriminates good from poor reading comprehenders, it should be able to classify the remaining 35% of participants whose data were not included in the development of each of the decision trees.

Note that the classification accuracy on “untrained” participants is slightly different each time the classifier is run through these two stages. This variability stems from factors that are randomized each time the classifier is run, such as which subsets of scalp sites and participants are sampled by different decision trees. In order to obtain a reliable measure of classification accuracy and weights for the top-10 scalp sites, we ran the classifier 1000 times and averaged the results to find the overall classification accuracy and the top-10 scalp sites that were consistently highly weighted.

To evaluate the statistical significance of classification, we ran the classifier 1000 additional times with the class labels (good vs. poor reading comprehenders) randomly scrambled each time. This yielded an estimate of the baseline accuracy of classification based on aspects of the electrophysiological data unrelated to reading comprehension performance, utilizing the same number of degrees of freedom of the original classification. Classification would be deemed statistically significant when the distribution of 1000 accuracy values for the correct-label classification was substantially shifted to higher accuracy relative to the distribution for the scrambled-label classification. We quantified this shift in three ways: (1) independent-groups *t*-test with individual accuracy values as the random effect, (2) effect size (the mean accuracy difference divided by the pooled standard deviation), and (3) proportions of hits and false positives for deciding whether a given accuracy derives from the correct-label or scrambled-label distribution based on an unbiased criterion.

As described earlier, we fed to the classifier the difference-CSDs at each 100-ms time bin baselined to the activity during the first 50 ms after target onset. CSD transformation was used to provide some advantages over raw ERPs in terms of reference independence and greater spatial resolution, the difference-CSDs were used to isolate comprehension specific processes over and above word-level processes, and baselining to the post-stimulus-onset activity was reasonable in the context of relatively rapid word presentation and prior ERP research in language processing. Nevertheless, to determine whether these particular data pre-processing choices were important, in several control runs of the classifier we fed the classifier the electrophysiological information in additional formats. To determine the relevance of CSD transformation, we fed the classifier the difference-ERPs without CSD transformation. To determine whether the use of difference waves was critical for isolating comprehension-specific processes, we attempted to classify good vs. poor reading comprehenders using CSD-transformed ERPs from either the scrambled condition or the comprehension condition. To determine whether the use of the ERPs to the target word was important, we attempted to classify good vs. poor comprehenders using difference-CSDs to non-target words. Finally, to determine the influence of the method of baselining on the extraction of electrophysiological signals relevant to comprehension, we fed the classifier CSD-transformed ERPs baselined to the entire epoch (including the 100 ms pre-stimulus period) as well as non-baselined data, while also including three additional time bins: −100 to 0, −50 to 50, and 0 to 100 ms.

It is possible that the entire ERP waveform might produce superior classification of good vs. poor comprehenders as compared to the values of the waveform at each time bin. We examined this possibility by entering difference waves at the eight 100-ms time bins into a single classifier to take into account the temporal profile of the waveform. This procedure did not improve classification performance for either the CSD-transformed or non-CSD-transformed data. We will thus report only the results of the time-bin-by-time-bin classification analysis.

## Results

### Behavioral results

**Figure 2:**
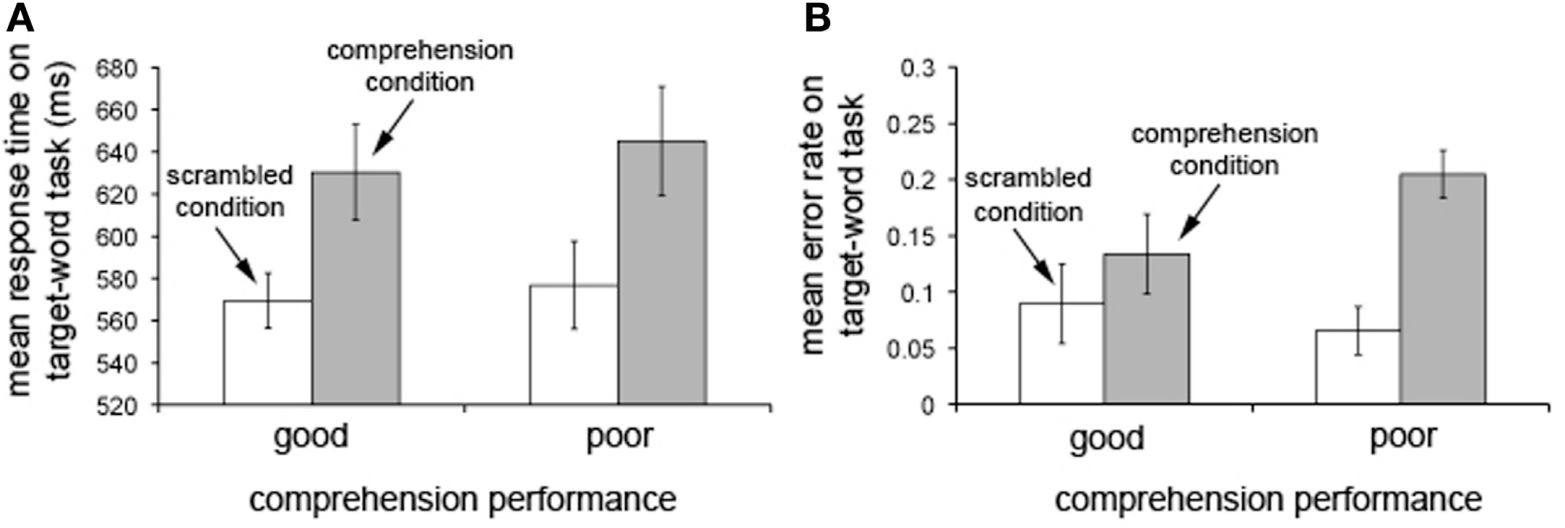
**Performance on the target-word detection task in the scrambled and comprehension conditions.** Response times **(A)** and error rates **(B)** on the target-word detection task in the scrambled condition (white bars) and the comprehension condition (gray bars) for individuals with good (*N* = 15; left bars) and poor (*N* = 13; right bars) reading comprehension. Note that target-detection performance is degraded in the comprehension condition relative to the scrambled condition for the poor comprehenders at least as much as for the good comprehenders. Error bars represent ±1 *SEM* (standard error of the mean). Data discussed in Mossbridge et al. (2013).

Patterns of target-word detection across conditions (Figure 2) verified that our good and poor reading comprehenders made equivalent effort at comprehending the extended text. If this were not the case, our electrophysiological analysis might provide a neural correlate of the amount of effort made to comprehend rather than individual differences in language processing associated with the ability to comprehend an extended text. Participants in both the good and poor comprehension groups slowed their responses [*F*_(1, 26)_ = 27.898, *p* < 10^−4^] and made more errors [*F*_(1, 26)_ = 9.373, *p* < 0.006] on target-word detection in the comprehension condition than in the scrambled condition. Importantly, there was neither a condition-by-group interaction nor a main effect of group for either response times [*F*_(1, 26)_ = 0.089, *n.s*. for interaction and *F*_(1, 26)_ = 0.168, *n.s*. for group effect] or error rates [*F*_(1, 26)_ = 2.691, *n.s*. for interaction and *F*_(1, 26)_ = 0.476, *n.s*. for group effect]. The lack of an interaction for both response times and error rates demonstrates that target-word detection performance was similarly degraded in the comprehension condition for both the good and poor comprehenders, suggesting that participants in both groups made an equivalent effort to perform the additional task of comprehending the story in the comprehension condition. Further, the lack of an interaction and the main effect for response times (i.e., similar response times in both conditions for the two groups) rules out the possibility that our electrophysiological analysis might reflect differences in motor preparation times instead of comprehension processes.

### Electrophysiological results

**Figure 3:**
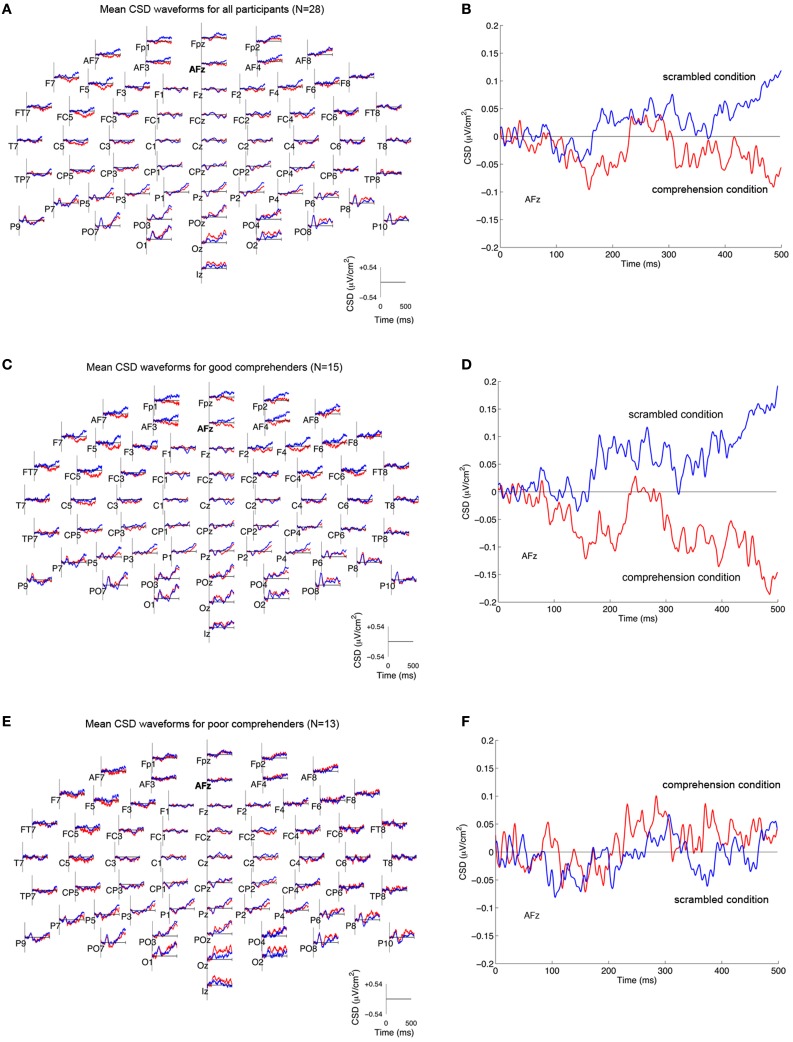
**Mean CSD-transformed ERP waveforms in the scrambled and comprehension conditions. (A)** CSD-transformed ERP waveforms (in μV/cm^2^) at each of 64 scalp sites time-locked to the onset of the target word (“and”) in the scrambled condition (blue) and the comprehension condition (red) for all participants (*N* = 28). Current sources are plotted as positive values, and current sinks as negative values. **(B)** CSD-transformed ERP waveforms at scalp site AFz for all participants. **(C,D)** CSD-transformed ERP waveforms for good comprehenders (*N* = 15) at all scalp sites **(C)** and at AFz **(D)**. **(E,F)** CSD-transformed ERP waveforms for poor comprehenders (*N* = 13) at all scalp sites **(E)** and at AFz **(F)**. The corresponding waveforms without CSD transformation are shown in Figure A1.

**Figure A1:**
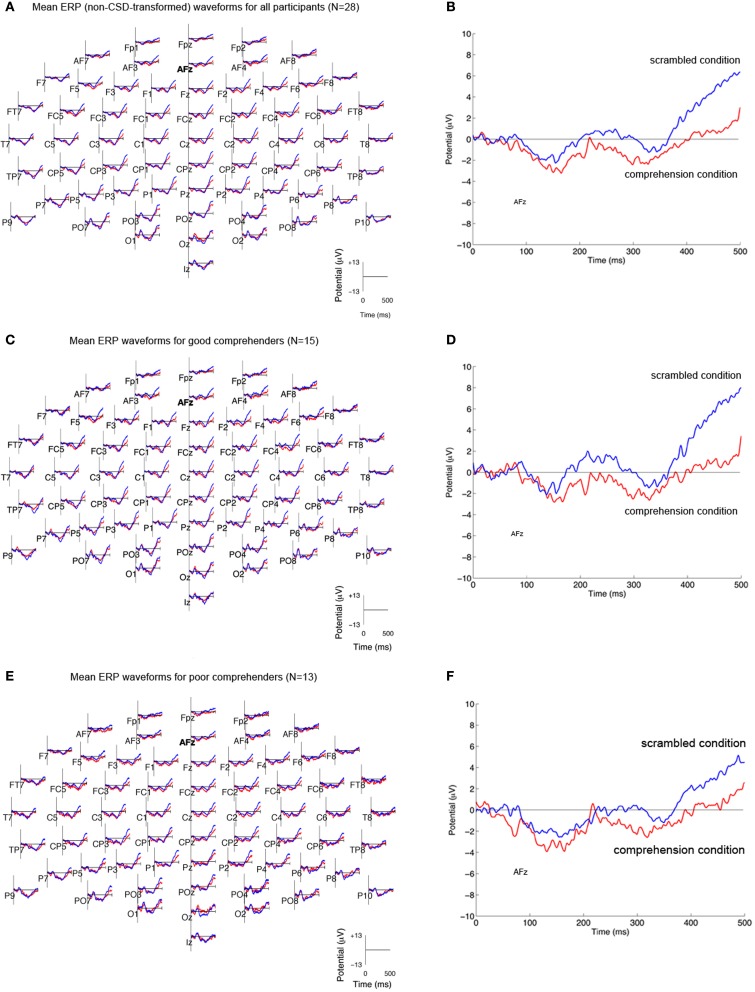
**Mean ERP (non-CSD-transformed) waveforms in the scrambled and comprehension conditions. (A)** ERP waveforms (nose referenced) at each of 64 scalp sites time-locked to the onset of the target word (‘and’) in the scrambled condition (blue) and the comprehension condition (red) for all participants (*N* = 28). On the Y-axis, the voltages (in μV) are plotted as positive values upward and negative values downward. **(B)** ERP waveforms at scalp site AFz for all subjects. **(C,D)** ERP waveforms for good comprehenders (*N* = 15) at all scalp sites **(C)** and at AFz **(D)**. **(E,F)** ERP waveforms for poor comprehenders (*N* = 13) at all scalp sites **(E)** and at AFz **(F)**.

We reasoned that neural activity specifically associated with comprehension processes should be reflected in the difference between CSD-transformed ERPs to the same target word in the scrambled and comprehension conditions (difference CSDs). CSD-transformed ERPs averaged across participants (*N* = 28) showed that the scrambled condition was marked by an increased current source at multiple sites relative to the comprehension condition (Figures 3A,B; Figure A1 gives the corresponding figure for non-CSD-transformed ERPs). When the mean CSD-transformed ERPs are plotted separately for the good and poor comprehenders, one can see that in the frontal sites for the good comprehenders, the positive-going wave for the scrambled condition diverges from the negative going wave for the comprehension condition (Figure 3C), whereas the two waves are less differentiated for the poor comprehenders (Figure 3E). This distinction between the comprehension groups is pronounced at AFz (Figures 3D,F), the site that was identified as critically informative in distinguishing good from poor comprehenders by our classification algorithm (see below). The divergence of the two waves appears to become maximal during 400–500 ms. Strong evoked activity is also seen in the posterior scalp sites, consistent with the fact that we used visually presented words as stimuli, but the posterior activity was not identified by our classification algorithm as informative for distinguishing good from poor comprehenders (see below).

**Figure 4:**
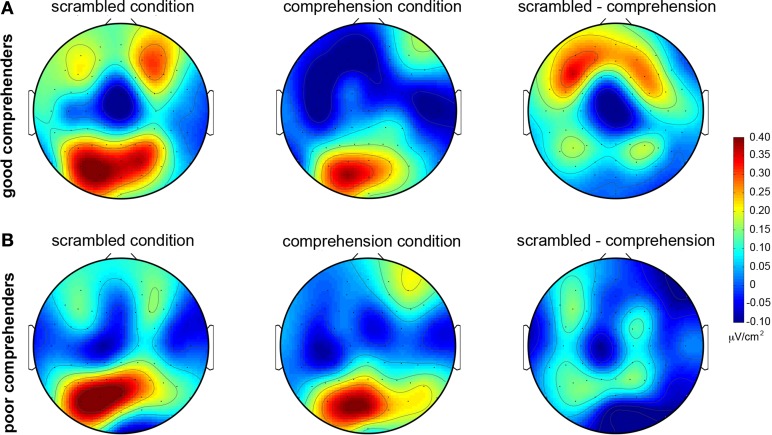
**Topographic maps of mean CSD-transformed ERPs during 400–500 ms post-target-word-onset in the scrambled and comprehension conditions. (A)** For good comprehenders. **(B)** For poor comprehenders. Warm and cool colors indicate current sources and sinks, respectively (see the color bar to the right of the figure). Left column: scrambled condition. Middle column: comprehension condition. Right column: difference map (scrambled condition minus comprehension condition). The corresponding topographic maps without CSD transformation are shown in Figure A2.

**Figure A2:**
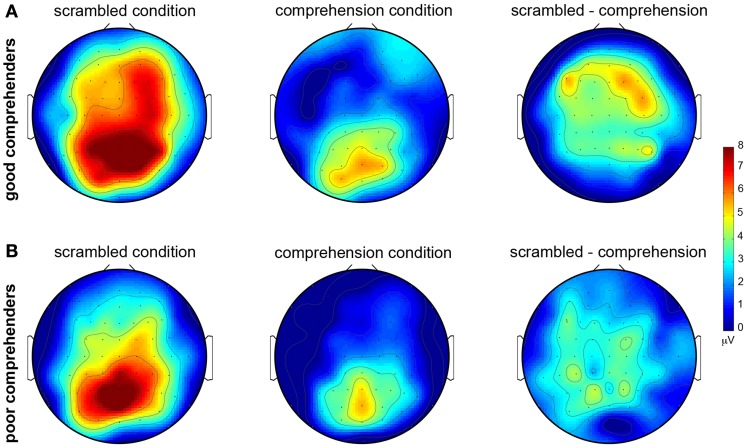
**Topographic maps of mean ERPs (non-CSD-transformed) during 400–500 ms post-target-word-onset in the scrambled and comprehension conditions. (A)** For good comprehenders. **(B)** For poor comprehenders. Warmer colors indicate greater positive voltages (see color bar to the right of the figure). Left column: scrambled condition. Middle column: comprehension condition. Right column: difference map (scrambled condition minus comprehension condition).

The topographic maps of CSD-transformed ERPs averaged over 400–500 ms confirm these observations (Figure 4); they show general posterior activity that is relatively undifferentiated with respect to condition (scrambled or comprehension) or comprehension performance (good or poor) (Figure A2 gives the corresponding figure for non-CSD-transformed ERPs). In contrast, the topographic map for the good comprehenders shows frontal activity indicating a current source (positive) in the scrambled condition turning into a current sink (negative) in the comprehension condition (Figure 4A), but the topographic map for the poor comprehenders reveals comparatively undifferentiated activity (Figure 4B). Although these CSD-transformed waveforms and topographic maps (Figures 3, 4) provide information about the time interval and scalp sites that may distinguish good from poor comprehenders, we used a pattern-classification algorithm to objectively determine the critical time interval and scalp sites for predicting individual differences in reading comprehension from these electrophysiological data.

**Figure 5:**
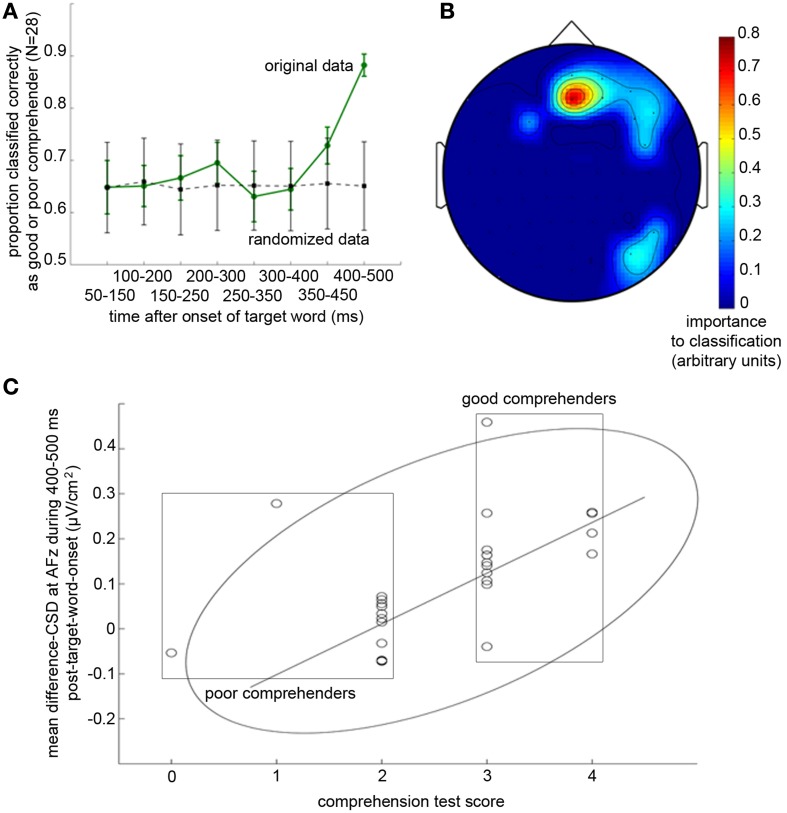
**Temporal and spatial profiles of difference-CSDs (CSD-transformed ERPs in the scrambled condition minus those in the comprehension condition) that predict individual differences in reading comprehension performance. (A)** Proportion of successful classification of participants into good and poor comprehenders based on difference-CSDs from eight overlapping 100-ms time bins. Solid green line shows the mean of 1000 classification results using the original data, and dotted gray line shows the mean of 1000 control classification results using the same data but with randomized labeling of good and poor comprehenders (see Methods). Error bars represent ±1 *SD* (standard deviation). It is apparent that the difference-CSDs during 400–500 ms following the onset of the target word are crucial for distinguishing good from poor comprehenders. **(B)** Topographic map showing the recording sites that were imporant for classifying good vs. poor comprehenders (warmer colors indicate greater importance). AFz is clearly the uniquely important scalp site. **(C)** Correlation between comprehension test scores and difference-CSDs at AFz during 400–500 ms following target word onset. The rectangles indicate the poor (left) and good (right) reading comprehenders, the ellipse indicates the boundary of the 95% confidence ellipse, and the line indicates the regression line without the three outliers.

Our classification algorithm identified the difference-CSDs to the target word from the 400–500 ms portion to be the most informative in predicting individual differences in reading comprehension, correctly classifying the participants as good or poor comprehenders with 88.3% accuracy [Figure 5A; *t*_(1998)_ = 85.2, *p* < 10^−30^, *d* = 4.35, proportions of hits and false-positives are 1.000 and 0.028, respectively; see Methods for explanation of these statistics]. Importantly, equivalent classification analyses using non-CSD transformed difference-ERPs, CSD-transformed ERPs from either the scrambled or comprehension condition alone (without taking the difference), difference-CSDs to the non-target words, and difference-CSDs with alternative baselining methods with the inclusion of pre-stimulus data, all failed to produce significant classification for any time period (see Methods). This suggests that CSD transformation, subtracting out the activity associated with word-level processing, and evaluating ERP signals to the target word relative to the initial 50 ms post-stimulus-onset period all contributed to the extraction of electrophysiological signals that distinguish good from poor reading comprehenders.

Interestingly, the classification algorithm virtually exclusively identified the difference-CSD at AFz (Figure 5B) as the critical activity related to reading comprehension. This result indicates a remarkable topographic specificity for the neural source of individual differences in extended-text comprehension, as our non-linear pattern classification algorithm could have identified any combination of the 64 scalp sites as being informative. To verify that the CSD index that the classifier identified based on binary classification of good vs. poor comprehenders (i.e., the difference-CSD from AFz averaged during 400–500 ms from target onset) indeed predicts individual differences, we computed the correlation between this CSD index and the reading comprehension test score. The correlation was robust, *r* = 0.803, *t*_(23)_ = 6.46, *p* < 10^−5^ [after removing 3 participants falling outside the 95% confidence ellipse, and *r* = 0.538, *t*_(26)_ = 3.25, *p* < 0.004 with the outliers included] (Figure 5C).

**Figure 6:**
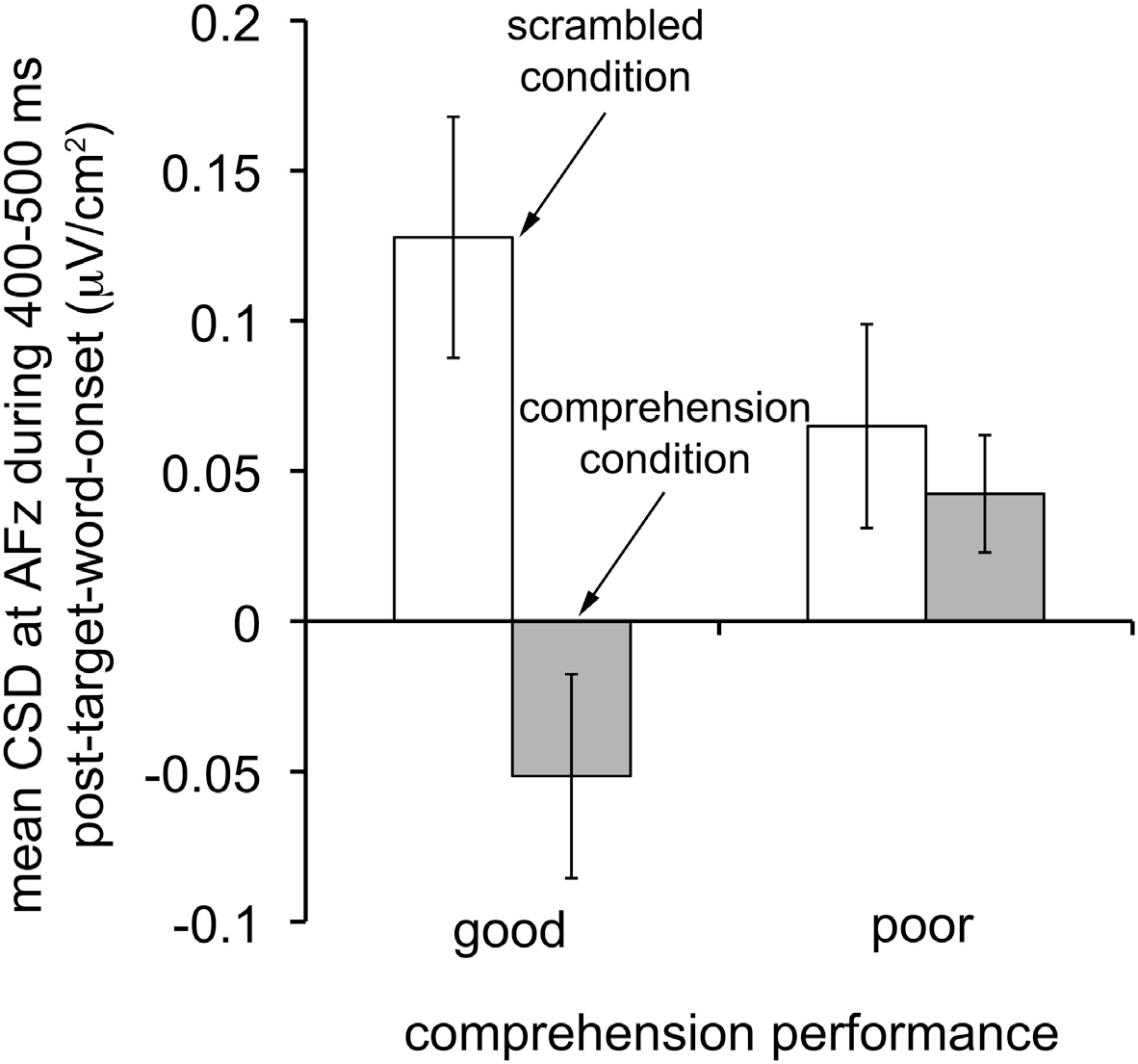
**Characteristics of CSD-transformed ERPs that distinguish good from poor reading comprehenders.** Mean CSD-transformed ERPs at scalp site AFz during the time period 400–500 ms after target word onset in the scrambled (white bars) and comprehension (gray bars) conditions for individuals with good (*N* = 15; left bars) and poor (*N* = 13; right bars) reading comprehension. Note that good comprehenders turned the frontal-midline current source into a current sink in the comprehension condition, whereas poor comprehenders did not. Error bars represent ±1 *SEM* (standard error of the mean).

To gain insights into how electrophysiological signatures associated with reading comprehension differ between good and poor comprehenders, we unpacked the CSD index based on ERP difference waves to examine how the critical CSD-transformed ERPs (from AFz at 400–500 ms) for the scrambled and comprehension conditions differed between good and poor comprehenders (Figure 6). For good comprehenders, the critical CSD-transformed ERPs differed dramatically between conditions, reflecting a current source in the scrambled condition and a current sink in the comprehension condition, *t*_(14)_ = 6.422, *p* < 10^−4^. In contrast, for the poor comprehenders the critical CSD-transformed ERPs did not differ between conditions, reflecting a current source in both conditions, *t*_(12)_ = 0.862, *n.s*. This pattern of results was confirmed by a significant condition-by-group interaction, *F*_(1, 26)_ = 16.471, *p* < 0.0005. Thus, superior ability in extended-text comprehension may be associated with underlying neural processes that flexibly turn a frontal-midline current source into a current sink when words need to be processed for comprehension as opposed to simple identification.

### Potential confound of eye movements

It is possible that individual differences in the stability of eye fixation might have contributed to the individual differences in comprehension performance if a systematic difference in eye movements contaminated our EEG signals (e.g., Dimigen et al., 2009). To verify that fixation stability did not systematically differ between our good and poor comprehenders, for each presentation of the target word for each participant, we determined the number of saccades during the corresponding 500-ms epoch based on the EOG activity. In the comprehension condition, the average numbers of saccades (per epoch) were *M* = 0.11 with *SD* = 0.12 (good comprehenders) vs. *M* = 0.12 with *SD* = 0.11 (poor comprehenders), and in the scrambled condition they were *M* = 0.09 with *SD* = 0.11 (good comprehenders) vs. *M* = 0.13 with *SD* = 0.11 (poor comprehenders), with no significant main effects or interaction (*F*'s < 1.342).

### Potential confound of condition order

Half of the participants performed the comprehension condition first, while the other half performed the scrambled condition first. Those who performed the scrambled condition first had a slight advantage when attempting to comprehend the story in the comprehension condition [*t*_(26)_ = 2.32, *p* < 0.04] likely because they had seen all the words of the story previously. We verified that the CSD index predicted reading comprehension over and above this order effect in two ways. We computed the correlation between the CSD index and comprehension score separately for participants who received each order. Both correlations were positive and relatively large, *r* = 0.922 for participants who received the scrambled condition first [*t*_(12)_ = 8.28, *p* < 0.000003, with no outliers beyond the 95% confidence ellipse], and *r* = 0.509 for participants who received the comprehension condition first [*t*_(9)_ = 1.78, *p* < 0.11, after removing outliers beyond the 95% confidence ellipse]. It is interesting to note that the CSD index was especially effective in predicting comprehension score (*r* > 0.90) when the comprehension condition was given after the scrambled condition. Although additional research is necessary to understand the source of this difference, a potential reason why the correlation was reduced for the participants who were given the comprehension condition first is that performing the comprehension condition might have made them engage some comprehension-related processes in the subsequent scrambled condition because the words reminded them of the story. Any engagement of comprehension-related processes in the scrambled condition would dilute the effectiveness of the CSD index that isolates comprehension-related processes by taking the difference between the scrambled and comprehension conditions. Importantly, we further verified that the CSD index predicted reading comprehension over and above the order effect by entering both the CSD index and order in a multiple regression model to predict comprehension score. As expected, the CSD index made a significant contribution [*t*_(26)_ = 3.59, *p* < 0.002] separately from order [*t*_(26)_ = 2.62, *p* < 0.02].

## Discussion

Electrophysiological signatures of word decoding and passage-level comprehension have been identified (Kutas and Hillyard, 1980, 1984; King and Kutas, 1995; VanPetten et al., 1997; Kutas and Federmeier, 2000; Maurer et al., 2007; Lau et al., 2008; Maurer and McCandliss, 2008; Coch and Mitra, 2010). Here we sought to identify a pattern of electrophysiological activity that predicted individual differences in extended-text comprehension. We compared ERPs to a function word “and” between a scrambled condition in which the words from a novel were presented in a random order and a comprehension condition in which the same words were presented in order and participants were asked to comprehend the story; a comprehension test was given at the end to assess the level of story comprehension. We reasoned that, whereas in the scrambled condition the ERP to “and” would only reflect the decoding and semantic processing of the word “and,” in the comprehension condition the ERP to “and” would additionally reflect comprehension-related processes such as reactivating the preceding concepts in anticipation of relating them to the upcoming concepts. We thus hypothesized that a spatiotemporal difference in ERPs between the two conditions would predict an individual's score on the comprehension test. We chose to analyze CSD-transformed ERPs because they provide reference-independent estimates of current sources and sinks with increased spatial sensitivity due to reduced influences from volume conduction (Hjorth, 1980; Kayser and Tenke, 2006; Tenke and Kayser, 2012).

Using a non-biased, data-driven pattern classification approach, we determined the spatiotemporal profile of difference-CSDs (the difference between CSD-transformed ERPs from the scrambled and comprehension conditions) that most reliably distinguished good from poor comprehenders. The identified critical spatiotemporal profile was surprisingly specific, focused at the frontal-midline scalp site, AFz, during the 400–500 ms period following the target word onset. Analyses of behavioral results suggested that this CSD index reflected the functioning of comprehension-related processes rather than reflecting amount of effort devoted to target detection, decision processing, or response preparation. Additional analyses showed that good comprehenders effectively turned the critical frontal-midline current from a source to a sink when comprehension was required, whereas poor comprehenders did not.

What comprehension processing does our CSD index reflect? Because single-word retrieval is necessary in both the scrambled and comprehension conditions in order to perform the target-word detection task, potential group differences in these processes are unlikely to have had a major influence on the CSD index. According to the simple model of reading (Gough and Tunmer, 1986), the other components of effective reading include integrating and maintaining word meanings over time. It is reasonable to assume that both integration and working memory processes were more strongly engaged in the comprehension condition than in the scrambled condition.

The CSD index may reflect the effectiveness of integration processes that generate expectations regarding upcoming words. These expectations would differ between the predictable comprehension condition and the unpredictable scrambled condition. To this point, we note that, although the CSD index baselined to the activity during the 50-ms post-stimulus-onset period strongly predicted comprehension performance, neither the CSD index baselined to the activity during the entire epoch nor the non-baselined CSD index predicted comprehension performance based on any time period including a 100-ms pre-stimulus period. Thus, our results could suggest that effective reading comprehension is associated with how expectations influence the unfolding of electrophysiological activity from initial (0–50 ms) to later (400–500 ms) processing of the target word.

Because working memory is necessary for semantic integration across words, the CSD index might also reflect the effectiveness of working memory processes. Various independent lines of research are consistent with this idea. Working-memory capacity is correlated with individual differences in reading comprehension (Daneman and Carpenter, 1980; Daneman and Merikle, 1996; Cain et al., 2004), individuals with comprehension-selective reading deficits have reduced working-memory capacity (Swanson et al., 2006; Cutting et al., 2009), training on working-memory tasks can improve reading comprehension (Chein and Morrison, 2010; Dahlin, 2011), and processing linguistic meaning at the level of sentences demands working-memory resources (VanPetten et al., 1997). Further, ERP components at frontal regions obtained while participants read sentences that imposed different working-memory loads partially distinguished how well-participants inferred the correct subject-verb relationship in each sentence (King and Kutas, 1995). It is thus possible that our CSD index might capture the electrophysiological signature of working memory processes that are particularly relevant to extended-text comprehension.

Note that the P300 and the N400 are two well-known ERP components (derived from non-CSD-transformed ERPs) that are present in the 300–700 ms time interval following visual stimuli, and that are influenced by expectation and working memory load (for reviews, see Kutas and Federmeier, 2000; Polich, 2007; Lau et al., 2008). The analyses of ERPs shown in the Appendix, however, indicate that the N400 was not apparent in our data, and while the P300 was apparent, it was neither associated with the CSD index nor predictive of comprehension performance. It is thus likely that our CSD index reflects integration and/or working memory processes that are different from, though potentially related to, those probed by the P300 or the N400.

What makes our CSD index sensitive to individual differences in extended-text comprehension? Our CSD index is based on the ERPs to the target word, “and.” Interestingly, when we similarly computed a CSD index but based on the ERPs to non-target words, the index no longer predicted comprehension performance. This suggests that either responding to a function word (such as “and”), responding to any target word, responding to a target word that is probabilistically rare in a text, or reading the word “and” irrespective of behavioral response, engages electrophysiological activity that is especially revealing of the effectiveness of comprehension-related processes. For example, it is possible that having participants respond to the target word might have helped to differentiate more effective vs. less effective comprehension processes by taxing the comprehension processes with a concurrent word-detection task. If this were the case our CSD index might provide a neural signature of interference-resistant language processing. However, any interference would have been minimal because the target word “and” was a highly familiar function word, it occurred only 2.4% of the time, and neither response times nor error rates to the target word significantly differed between our good and poor comprehenders. Future research needs to investigate these possibilities by manipulating the linguistic category of target words and their frequency (including having no target task).

It is important to point out that without CSD transformation, a similarly computed ERP index does not predict comprehension performance (see Appendix). Thus, the increased spatial resolution (via reduced influence of volume conduction) achieved by CSD transformation helped to localize the comprehension-specific electrophysiological activity on the scalp. It is possible that taking the second spatial derivative of scalp potentials is particularly effective because the electrophysiological activity that distinguishes good from poor reading comprehenders is most effectively distinguished as current sources and sinks. However, CSD transformation is not the only method for identifying ERP scalp topographies with enhanced spatial resolution. For example, the method of second-order blind source separation (Tang et al., 2005) might have produced equivalently or more effective spatiotemporal components. Alternatively, a method called “topographic ERP analysis” enhances spatial resolution by transforming scalp potentials to electric field configurations, and it further facilitates the identification of behaviorally relevant field configurations by identifying “microstates” that are stable for about 20–500 ms, allowing for comparison of those microstates between experimental conditions (for review, see Murray et al., 2008). It is possible that if microstates distinguishing between the scrambled and comprehension conditions are fed to the classifier, we might obtain a field configuration that predicts comprehension performance with greater accuracy. However, substantial improvement is unlikely because our CSD index is already strongly correlated with comprehension scores (*r* = 0.803 when three outliers are removed) and the critical scalp site is surprisingly focal, suggesting that CSD transformation effectively isolated comprehension-related electrophysiological activity in our experimental paradigm.

In summary, our results demonstrate that the modulation of frontal-midline electrophysiological responses to target words due to attempting to comprehend a story distinguishes good from poor comprehenders with a high degree of accuracy. This robust electrophysiological signature of comprehension-specific processing may lead to a new means to investigate the neural substrates of comprehension, to continuously track comprehension while reading, and may potentially aid the remediation of disabilities that compromise comprehension.

### Conflict of interest statement

The authors declare that the research was conducted in the absence of any commercial or financial relationships that could be construed as a potential conflict of interest.

## Figures and Tables

**Figure A3:**
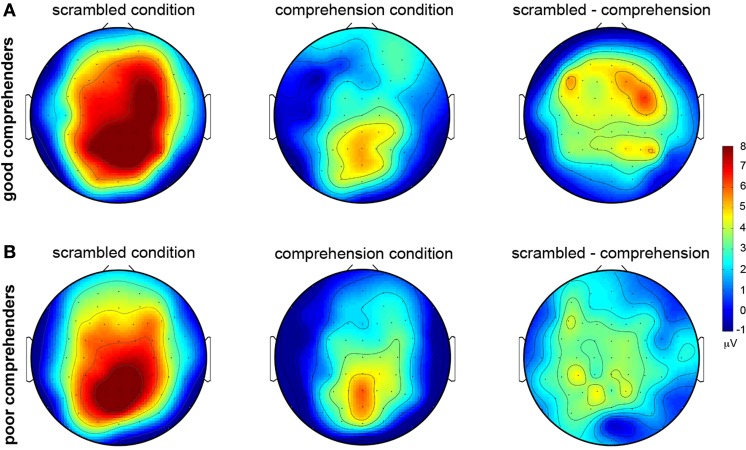
**Topographic maps of mean P300 difference waves (non-CSD-transformed ERPs to targets minus those to non-targets) during 400–500 ms in the scrambled and comprehension conditions. (A)** For good comprehenders. **(B)** For poor comprehenders. Warmer colors indicate more positive voltage differences, cooler colors indicate more negative voltage differences (see color bar to the right of the figure). Left column: scrambled condition. Middle column: comprehension condition. Right column: difference map (scrambled condition minus comprehension condition).

## Appendix

### Supplemental analyses

The predictability of the upcoming word was much higher in the comprehension than the scrambled condition, and the comprehension condition required more attention and/or working memory resources (to understand the story for the later comprehension test) than the scrambled condition. Thus, our CSD index of extended-text comprehension may reflect expectation and/or working memory processes. The P300 and the N400 are two well-known ERP components (derived from non-CSD-transformed ERP analyses) that have been shown to reflect electrophysiological responses to variations in expectation and working memory load. The N400 typically peaks in response to target stimuli around 400–500 ms, while the peak of the P300 varies between 300 and 700 ms, depending on the time necessary for stimulus evaluation (for reviews, see Kutas and Federmeier, 2000; Polich, 2007; Lau et al., 2008). The P300 becomes more positive when rare or unexpected events occur and when working-memory load is lower (Watter et al., 2001); thus, the P300 would be expected to be more positive in the scrambled than the comprehension condition. In line with this idea, the ERP activity during 400–500 ms is more positive in the scrambled than the comprehension condition at the posterior scalp electrodes relevant to the P300 (see Figure A1 for ERP waveforms and Figure A2 for the ERPs averaged for the 400–500 ms period).

As for the N400, the ERPs were positively trending (positively sloped as a function of time) during 300-500 ms with no clear indication of negatively trending potentials in that interval (see Figure A1). Large P300 effects during this interval are likely, given the low probability of target events (2.4% of the words). Thus, the paradigm we used does not allow the isolation of the N400, so that a relationship between our CSD index and the N400 may be possible, though it is not apparent here.

It may seem plausible that our frontal-midline CSD comprehension index reflects differences in the P300 between the scrambled and comprehension conditions, but upon further examination the data do not support this interpretation. The P300 is conventionally revealed as the late positive peak in the difference wave obtained by subtracting ERPs to non-target stimuli (here, all words other than “and”) from ERPs to target stimuli (here, “and”). Applying this analysis to the present data reveals posterior positive peaks indicative of the P300 in both the scrambled and comprehension conditions for both good and poor comprehenders during 400–500 ms post-word-onset (Figure A3).

To examine the relationship between the P300 and comprehension performance as well as between the P300 and our CSD comprehension index, we averaged the P300 difference wave across its peak scalp site (Pz) and four surrounding electrodes (CPz, P1, P2, and POz) separately for both the scrambled and comprehension conditions, then performed a correlation analysis. None of the correlations was significant (comprehension condition P300 vs. comprehension performance: *r* = −0.245, *n.s*.; comprehension condition P300 vs. CSD comprehension index: *r* = 0.234, *n.s*.; scrambled condition P300 vs. comprehension performance: *r* = −0.268, *n.s*.; scrambled condition P300 vs. CSD comprehension index: *r* = 0.056, *n.s*.). Further, after creating a condition-difference P300 measure by subtracting the P300 difference wave for the comprehension condition from the P300 difference wave for the scrambled condition (at the same five posterior electrodes), there were no significant correlations between this condition-difference P300 measure with either reading comprehension performance or our CSD comprehension index (condition-difference P300 vs. comprehension performance: *r* = −0.110, *n.s*.; condition-difference P300 vs. CSD comprehension index: *r* = −0.156, *n.s*.). Finally, there is some frontal activity accompanying the P300 that appears to be different between good and poor reading comprehenders. This P300-related activity is especially apparent in good but not poor comprehenders at scalp sites F2 and FC4 (warmest colors in Figure A3, right column). However, this frontal condition-difference P300-related activity, averaged across scalp sites F2 and FC4, is again not significantly correlated with either reading comprehension performance or our CSD comprehension index (frontal condition-difference P300-related activity vs. comprehension performance: *r* = −0.057, *n.s*.; frontal condition-difference P300-related activity at vs. CSD comprehension index: *r* = −0.041, *n.s*.).

Taken together, it is plausible that our CSD index of extended-text comprehension may be influenced by condition differences in expectancy and/or working-memory load, as are the P300 and N400. However, we found no evidence of any reliable relationship between our CSD comprehension index and the P300 or N400, and only the CSD comprehension index was reliably correlated with extended-text comprehension performance.

### Text used in the comprehension and scrambled conditions

The text below (starting with “This text is taken … ”) was presented one word at a time in a randomized order (in the single-task condition) or in the correct order (in the dual-task condition).

This text is taken from “Doctor Pascal” by Emile Zola

In the heat of the glowing July afternoon, the room, with blinds carefully closed, was full of a great calm. From the three windows, through the cracks of the old wooden shutters, came only a few scattered sunbeams which, in the midst of the obscurity, made a soft brightness that bathed surrounding objects in a diffused and tender light. It was cool here in comparison with the overpowering heat that was felt outside, under the fierce rays of the sun that blazed upon the front of the house.

Standing before the press which faced the windows, Dr. Pascal was looking for a paper that he had come in search of. With doors wide open, this immense press of carved oak, adorned with strong and handsome mountings of metal, dating from the last century, displayed within its capacious depths an extraordinary collection of papers and manuscripts of all sorts, piled up in confusion and filling every shelf to overflowing. For more than 30 years the doctor had thrown into it every page he wrote, from brief notes to the complete texts of his great works on heredity. Thus, it was that his searches here were not always easy. He rummaged patiently among the papers, and when he at last found the one he was looking for, he smiled.

For an instant longer he remained near the bookcase, reading the note by a golden sunbeam that came to him from the middle window. He himself, in this dawnlike light, appeared, with his snow-white hair and beard, strong and vigorous; although he was near sixty, his color was so fresh, his features were so finely cut, his eyes were still so clear, and he had so youthful an air that one might have taken him, in his close-fitting, maroon velvet jacket, for a young man with powdered hair.

“Here, Clotilde,” he said at last, “you will copy this note. Ramond would never be able to decipher my diabolical writing.”

And he crossed the room and laid the paper beside the young girl, who stood working at a high desk in the embrasure of the window to the right.

“Very well, master,” she answered.

She did not even turn round, so engrossed was her attention with the pastel which she was at the moment rapidly sketching in with broad strokes of the crayon. Near her in a vase bloomed a stalk of hollyhocks of a singular shade of violet, striped with yellow. But the profile of her small round head, with its short, fair hair, was clearly distinguishable; an exquisite and serious profile, the straight forehead contracted in a frown of attention, the eyes of an azure blue, the nose delicately molded, the chin firm. Her bent neck, especially, of a milky whiteness, looked adorably youthful under the gold of the clustering curls. In her long black blouse she seemed very tall, with her slight figure, slender throat, and flexible form, the flexible slenderness of the divine figures of the Renaissance. In spite of her 25 years, she still retained a childlike air and looked hardly eighteen.

“And,” resumed the doctor, “you will arrange the press a little. Nothing can be found there any longer.”

“Very well, master,” she repeated, without raising her head; “presently.”

Pascal had turned round to seat himself at his desk, at the other end of the room, before the window to the left. It was a plain black wooden table, and was littered also with papers and pamphlets of all sorts. And silence again reigned in the peaceful semi-obscurity, contrasting with the overpowering glare outside. The vast apartment, a dozen meters long and six wide, had, in addition to the press, only two bookcases, filled with books. Antique chairs of various kinds stood around in disorder, while for sole adornment, along the walls, hung with an old *salon* Empire paper of a rose pattern, were nailed pastels of flowers of strange coloring dimly visible. The woodwork of three folding-doors, the door opening on the hall and two others at opposite ends of the apartment, the one leading to the doctor's room, the other to that of the young girl, as well as the cornice of the smoke-darkened ceiling, dated from the time of Louis XV.

An hour passed without a sound, without a breath. Then Pascal, who, as a diversion from his work, had opened a newspaper–Le Temps–which had lain forgotten on the table, uttered a slight exclamation:

“Why! your father has been appointed editor of the *Epoque*, the prosperous republican journal which has the publishing of the papers of the Tuileries.”

This news must have been unexpected by him, for he laughed frankly, at once pleased and saddened, and in an undertone he continued:

“My word! If things had been invented, they could not have been finer. Life is a strange thing. This is a very interesting article.”

Clotilde made no answer, as if her thoughts were a hundred leagues away from what her uncle was saying. And he did not speak again, but taking his scissors after he had read the article, he cut it out and pasted it on a sheet of paper, on which he made some marginal notes in his large, irregular handwriting. Then he went back to the press to classify this new document in it. But he was obliged to take a chair, the shelf being so high that he could not reach it notwithstanding his tall stature.

On this high shelf a whole series of enormous bundles of papers were arranged in order, methodically classified. Here were papers of all sorts: sheets of manuscript, documents on stamped paper, articles cut out of newspapers, arranged in envelopes of strong blue paper, each of which bore on the outside a name written in large characters. One felt that these documents were tenderly kept in view, taken out continually, and carefully replaced; for of the whole press, this corner was the only one kept in order.

When Pascal, mounted on the chair, had found the package he was looking for, one of the bulkiest of the envelopes, on which was written the name “Saccard,” he added to it the new document, and then replaced the whole under its corresponding alphabetical letter. A moment later he had forgotten the subject, and was complacently straightening a pile of papers that were falling down. And when he at last jumped down off the chair, he said:

“When you are arranging the press, Clotilde, don't touch the packages at the top; do you hear?”

“Very well, master,” she responded, for the third time, docilely.

He laughed again, with the gaiety that was natural to him.

“That is forbidden.”

“I know it, master.”

And he closed the press with a vigorous turn of the key, which he then threw into a drawer of his writing table.

### Comprehension test

Each question was scored as correct if all correct answers were circled and all incorrect answers were not circled, and incorrect otherwise. Thus, the maximum possible comprehension score was 4, the minimum possible comprehension score was 0, and the score expected by chance was less than 1 (0.27 correct). The correct answers are bolded below.

Please circle the answer that is most appropriate, given your understanding of the text you just read on the screen. More than one answer can be appropriate for each question.

1. What is the relationship between Dr. Pascal and Clotilde? (please circle all that apply)
  - She is his wife.
  - **She is his niece.**
  - **She is his secretary.**
  - She is a lover.
2. What is the occupation of Dr. Pascal? (please circle all that apply)
  - **He is a scientist or doctor.**
  - He is a mathematician.
  - **He studies heredity.**
  - He is a hunter.
3. How does the room they are in differ from outside? (please circle all that apply)
  - It is hotter than outside.
  - **It is colder than outside.**
  - It is brighter than outside.
  - **It is darker than outside.**
4. Why can't Dr. Pascal reach the top shelf? (please circle all that apply)
  - He is too short.
  - **It's really high.**
  - The glasses and knick-knacks get in his way.
  - He has a broken leg.
